# Extension of the bayesian alphabet for genomic selection

**DOI:** 10.1186/1471-2105-12-186

**Published:** 2011-05-23

**Authors:** David Habier, Rohan L Fernando, Kadir Kizilkaya, Dorian J Garrick

**Affiliations:** 1 Department of Animal Science and Center for Integrated Animal Genomics, Iowa State University, Ames, IA 50011, USA; 2 Department of Animal Science, Adnan Menderes University, Aydin 09100, Turkey; 3 Institute of Veterinary, Animal & Biomedical Science, Massey University, Palmerston North, New Zealand

## Abstract

**Background:**

Two Bayesian methods, BayesC*π *and BayesD*π*, were developed for genomic prediction to address the drawback of BayesA and BayesB regarding the impact of prior hyperparameters and treat the prior probability *π *that a SNP has zero effect as unknown. The methods were compared in terms of inference of the number of QTL and accuracy of genomic estimated breeding values (GEBVs), using simulated scenarios and real data from North American Holstein bulls.

**Results:**

Estimates of *π *from BayesC*π*, in contrast to BayesD*π*, were sensitive to the number of simulated QTL and training data size, and provide information about genetic architecture. Milk yield and fat yield have QTL with larger effects than protein yield and somatic cell score. The drawback of BayesA and BayesB did not impair the accuracy of GEBVs. Accuracies of alternative Bayesian methods were similar. BayesA was a good choice for GEBV with the real data. Computing time was shorter for BayesC*π *than for BayesD*π*, and longest for our implementation of BayesA.

**Conclusions:**

Collectively, accounting for computing effort, uncertainty as to the number of QTL (which affects the GEBV accuracy of alternative methods), and fundamental interest in the number of QTL underlying quantitative traits, we believe that BayesC*π *has merit for routine applications.

## Background

High-density single nucleotide polymorphisms (SNPs) covering the whole genome are available in animal and plant breeding to estimate breeding values. First, individuals having SNP genotypes and trait phenotypes are used to estimate SNP effects (training), and then genomic estimated breeding values (GEBVs) are obtained for every genotyped individual using those effects. Currently, the number of SNP genotypes per individual amounts to tens of thousands, but, owing to the rapid advances in genomics, it will soon exceed millions at comparable costs. The statistical challenge is to estimate SNP effects in a situation where the number of training individuals is much smaller than the vast number of SNPs. For this purpose, Meuwissen et al. [1] presented two hierarchical Bayesian models, termed BayesA and BayesB, that are discussed extensively in animal and plant breeding research (e.g., [2-6]). The reason for their popularity is that their implementation as single site locus sampler is straightforward, computing time is reasonable, and both simulations [1,7,8] and real data analyses [9,10] have shown that linkage disequilibrium (LD) between SNPs and quantitative trait loci (QTL) is exploited better than with least-squares or ridge-regression; hence, accuracies of GEBVs were higher for these Bayesian methods. Gianola et al. [11] pointed to statistical drawbacks of BayesA and BayesB that center around the prior for SNP effects. A priori, a SNP effect is zero with probability *π*, and normally distributed having mean zero and a locus-specific variance with probability (1-*π*). This locus-specific variance has a scaled inverse chi-square prior with few degrees of freedom and a scale parameter, , that is often derived from an assumed additive-genetic variance under certain genetic assumptions [11,12]. It can be shown that the full-conditional posterior of a locus-specific variance has only one additional degree of freedom compared to its prior regardless of the number of genotypes or phenotypes. This conflicts with the concept of Bayesian learning, and as a consequence, shrinkage of SNP effects depends strongly on  as detailed by [11]. This problem becomes even more important with increasing SNP density as shown later. There are at least two possibilities to overcome this drawback: First, a single effect variance that is common to all SNPs is used instead of locus-specific variances. Then, as shown later, the influence of  is smaller. Second, the scale parameter of the inverse chi-square prior for locus-specific variances is treated as an unknown with its own prior. The first strategy is referred to as BayesC in the following and the second as BayesD.

Another drawback of BayesA and BayesB is that the probability *π *that a SNP has zero effect is treated as known. In BayesA, *π *= 0 so that all SNPs have non-zero effect, whereas in BayesB, *π *> 0 to accommodate the assumption that many SNPs have a zero effect. The shrinkage of SNP effects is affected by *π*, and thus should be treated as an unknown being inferred from the data. In the following, *π *is treated as an unknown in BayesC and BayesD, which will be referred to as BayesC*π *and BayesD*π*, respectively. Finally, the question arises how the estimated *π *is related to the number of QTL.

The objective of this study was to present two Bayesian model averaging methods that address the drawback of BayesA and BayesB regarding the impact of  on shrinkage of SNP effects, and treat *π *as an unknown by using BayesC*π *and BayesD*π*. Simulations were conducted to analyze estimates of *π *for the ability to infer the number of QTL depending on the genetic architecture of a quantitative trait and training data size. Field data from North American Holstein bulls were used to estimate *π *for milk production traits, and to compare accuracies of GEBVs obtained by BayesA, BayesB, BayesC*π*, BayesD*π*, and ridge-regression. Cross-validations were applied in a setting where the additive-genetic relationships between training and validation bulls were low so that the accuracies of GEBVs were dominated by LD information. This criterion reveals the potential of genomic selection better than accuracy obtained by using training data sets that contain close relatives of validation bulls such as parents, full and half sibs. The reason is that future selection candidates in cattle breeding programs may not have close relatives in training when genomic selection is applied early in life [9].

## Methods

### Statistical Model

The general statistical model can be written as

where ***y ***is an *N *× 1 vector of trait phenotypes, **X **is an incidence matrix of the fixed effects in ***β***, ***u ***is a vector with polygenic effects of all individuals in the pedigree, *K *is the number of SNPs, ***z****_k _*is an *N *× 1 vector of genotypes at SNP *k*, *a_k _*is the additive effect of that SNP, and ***e ***is a vector of residual effects. In this study, the only fixed effect in ***β ***was the overall mean *μ*, and SNP genotypes were coded as the number of copies of one of the SNP alleles, i.e., 0, 1 or 2.

### Prior specifications

The prior for *μ *was a constant; the prior for ***u***|**A**,  was normal with mean zero and variance , where **A **is the numerator-relationship matrix and  is the additive-genetic variance not explained by SNPs. The prior for *a_k _*depends on the variance, , and the prior probability *π *that SNP *k *has zero effect:(1)

The models of this study differed in their specifications for *π *and . In BayesA, BayesB and BayesD*π*,  denotes that each SNP has its own variance. Each of these variances has a scaled inverse chi-square prior with degrees of freedom *ν_a _*and scale , and thus with probability (1-*π*) the marginal prior of *a_k_*|*ν_a_*,  is a univariate student's t-distribution, . This is the model hierarchy proposed by [1], where  was derived here from the expected value of a scaled inverse chi-square distributed random variable, ; hence,(2)

where *ν_a _*was 4.2 as in [1], and  is the variance of the additive effect for a randomly sampled locus, which can be related to the additive-genetic variance explained by SNPs, , as(3)

where *p_k _*is the allele frequency of SNP *k *[11-13]. BayesC*π *and BayesD*π *are constructed as follows to address the lack of Bayesian learning in BayesA and BayesB.

In BayesC*π*, , i.e., the priors of all SNP effects have a common variance, which has a scaled inverse chi-square prior with parameters *ν_a _*= 4.2 and , where  is derived as for BayesA and BayesB. As a result, the effect of a SNP fitted with probability (1-*π*) comes from a mixture of multivariate student's t-distributions, . For example, assume that only 3 SNPs are used in the analysis, resulting in 4 possible models in which the effect of SNP 1, say, is not zero (Table 1). Each of these models has a different multivariate t-prior, where the univariate t-distribution is regarded here as a special case of the multivariate distribution. Thus, across the 4 models, the effect of SNP 1 comes from a mixture of multivariate t-distributions.

**Table 1:** Model configurations in which SNP 1 has non-zero effect for an example using three SNPs in the analysis

	Model			
	
SNP effect	1	2	3	4
*a*_1_	≠0	≠0	≠0	≠0
*a*_2_	≠0	0	≠0	0
*a*_3_	≠0	≠0	0	0

In BayesD*π*, the degrees of freedom for the scaled inverse chi-square prior of the locus-specific variances, *ν_a_*, are treated as known with a value of 4.2 as in all other models, but the scale parameter, , is treated as an unknown with Gamma(1,1) prior. Thus, for a SNP fitted with probability (1-*π*), its effect comes from a mixture of univariate student's t-distributions. In this case, the mixture is due to treating  as unknown with a gamma prior.

The other parameter that must be specified for the prior of *a_k _*in (1) is *π*, which is treated as known with *π *= 0 for BayesA and, in this paper, with *π *= 0.99 for BayesB. In BayesC*π *and BayesD*π*, in contrast, *π *is treated as an unknown with uniform(0,1) prior.

The prior for the residual effects is normal with mean zero and variance , and the priors for  and  are scaled inverse chi-square with arbitrarily small value of 4.2 for the degrees of freedom, and scale parameters  and , respectively. These scale parameters were derived by the formula , where  is the *a priori *value of  or .

## Inference of model parameters

Two Markov Chain Monte Carlo (MCMC) algorithms were implemented to infer model parameters: one for BayesA, BayesB, and BayesD*π *and the other one for BayesC*π*. The differences between these two algorithms result from how the variances of SNP effects are modeled and lead to different strategies for including a SNP in the model.

### Algorithm for BayesA, BayesB and BayesD*π*

BayesA is a special case of BayesB with *π *= 0. The variables *μ*, *a_k_*, ***u***, , , as well as  and *π *of BayesD*π *are sampled by Gibbs-steps using their full-conditional posteriors, whereas the decision to fit SNP *k *into the model and the value of its locus-specific variance, , are sampled by a Metropolis-Hastings (MH) step. In contrast to Meuwissen et al. [1], who implemented BayesA using Gibbs sampling, BayesA is implemented here as BayesB with *π *= 0 and a reduced number of MH steps.

The MH step used in this study differs from that described for BayesB in [1]. In their implementation, the candidate for  is sampled from the scaled inverse chi-square prior with probability (1 - *π*), whereas a model without SNP *k *is proposed with probability *π*. In the latter case both *a_k _*and  are equal to zero. The acceptance probability for the candidate sample in iteration *t *from the currently accepted variance, , to the candidate value, , is

where  and  denote densities of the data model given  and , respectively, and all other model parameters denoted by ELSE as in Sorensen and Gianola [14], except for *a_k _*which is integrated out here. Values of *π *close to 1 lead to candidate samples that are mostly 0, and thus in poor mixing. To increase the probability of non-zero candidates, the MH step is repeated 100 times in each iteration of the MCMC algorithm.

The proposal distribution for  used here is different from the prior. Regardless of *π*, the candidate for  is sampled with probability 0.5 from a scaled inverse chi-square, and with probability 0.5 from a point mass on zero, which reduces the number of MH steps required for mixing. The number of MH steps used here was 10. Further, the scale parameter  of the candidate is chosen depending on whether SNP *k *was in the model in the previous iteration *t *- 1 or not, i.e., whether  or equals to zero:

The acceptance probability is

where the prior for  is

and its proposal is

This proposal is expected to have better mixing than that of [1] for extreme values of *π*. The acceptance probability is equivalent to equation 2.4 in Godsill (2001) [15].

After  has been updated, *a_k _*is sampled from(4)

where  and . After  and *a_k _*have been updated for all *K *SNPs, the polygenic effects in ***u ***are sampled by the technique of [16] as described in [14] using an iterative algorithm to solve the mixed model equations;  is sampled from a scaled inverse chi-square with degrees of freedom  and scale , where *n_u _*is the number of individuals in the pedigree;  is sampled from a scaled inverse chi-square with  and , where *n *is the number of training individuals. In BayesD*π*,  is sampled from a gamma with shape  and scale , where *m*^(*t*) ^is the number of SNPs fitted in the model for iteration *t*. The parameters of this gamma posterior show that information from all loci contributes to the posterior of the unknown scale parameter and therefore through it to the posteriors of the locus-specific variances. Finally, *π *is drawn from Beta(*K *- *m*^(*t*) ^+ 1, *m*^(*t*) ^+ 1). The starting value for *π *was 0.5.

### Algorithm for BayesC*π*

The MCMC algorithm for BayesC*π *consists of Gibbs steps only, where those for *μ*, ***u***, , , and *π *are identical to those in BayesD*π*. In contrast, the decision to include SNP *k *in the model depends on the full-conditional posterior for the indicator variable *δ_k_*, which is introduced for this very purpose. This indicator variable equals 1 if SNP *k *is fitted to the model and is zero otherwise. Following general Bayesian rules, the full-conditional posterior probability that *δ_k _*= 1 is

where *p*(***y***|ELSE) = *p*(***y***|*δ_k _*= 0, ELSE)*p*(*δ_k _*= 1|*π*);  denotes the density of the data model given that SNP *k *is fitted with common effect variance  and the currently accepted values of all other parameters, *p*(***y***|*δ_k _*= 0, ELSE) is the density of the data model without SNP *k*, *p*(*δ_k _*= 0|*π*) = *π *is the prior probability that SNP *k *has zero effect, and correspondingly *p*(*δ_k _*= 1|*π*) = 1 - *π*. The posterior for *a_k _*is identical to (4) except that  replaces the locus-specific variance in *c_k _*so that . The common effect variance is sampled from a full-conditional posterior, which is a scaled inverse chi-square with degrees of freedom  and scale , where *m*^(*t*) ^is the number of SNPs fitted with non-zero effect in iteration *t*.

The starting value for *π*, *π*_0_, determines  as can be seen from equations (2) and (3). However,  can affect to what extent *π *is used to shrink SNP effects, hence the estimate of *π*. As  increases with *π*_0_, less shrinkage is expected through , but shrinkage can be increased with larger *π *values, which can be regarded as a compensation for the lower shrinkage through . To examine the effect of *π*_0 _in BayesC*π*, results are given for *π*_0 _equal to 0.5, 0.8 and 0.95. The degrees of freedom of the scaled inverse chi-square prior, *ν_a_*, also determine  through formula (2), and thus can affect *π *estimates. However, in this study *ν_a _*was not varied, but held constant at 4.2.

### Impact of  on shrinkage in BayesC*π *compared to BayesA and BayesB

The parameters of this full-conditional distribution can be used to contrast the impact of  on shrinkage in BayesC*π *compared to that in BayesA and BayesB. In the latter, the posterior of the locus-specific variance of SNP *k *is a scaled inverse chi-square distribution with degrees of freedom  and scale [11]. That is, that posterior has only one more degree of freedom than the prior. In contrast, the full-conditional of the posterior of the common effect variance in BayesC*π *will have more degrees of freedom when *m*^(*t*) ^> 1 and the scale is less influenced by  and more a function of the information contained in the data through .

The impact of  on the shrinkage of SNP effects, especially for BayesA, increases with SNP density. The scale parameter, , decreases with increasing number of SNPs in the analyses due to , which depends on *π*. Hence, small SNP effects are regressed more towards zero than with a smaller number of SNPs in the model. Consider a chromosomal segment where a QTL is surrounded by many SNPs that are all in LD with the QTL. In the worst case, all these SNPs are collinear, which might occur for low effective population sizes. The QTL effect, even if large, will be distributed to all SNPs such that each SNP effect is small. As these effects are strongly regressed towards zero, the QTL effect can be completely lost.

### Software implementation

These Bayesian model averaging methods were implemented in *GenSel *software [17] and are available for web-based analysis of genomic data. It is accessible through BIGS.ansci.iastate.edu, and a user manual is attached to this manuscript in additional file 1.

## Simulations

**Figure 1:**
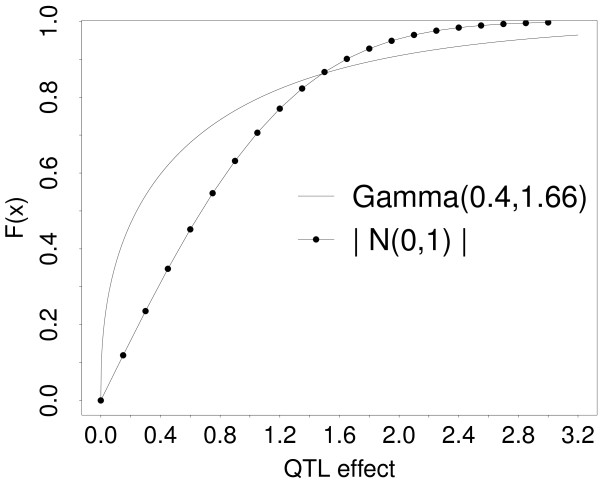
**Cumulative distribution functions, F(x), of the distributions used to sample QTL effects: Gamma with shape 0.4 and scale 1.66 and absolute standard normal**.

Simulations were conducted to analyze estimates of *π *from BayesC*π *and BayesD*π *depending on the genetic architecture of an additive quantitative trait, and training data size. Two types of scenarios were simulated in this study. The first was an ideal scenario in which all loci were in mutual linkage equilibrium and genotypes of both SNPs and QTL were available for training and validation. The true value of *π *is the number of QTL divided by the total number of loci in this analysis. The second was a realistic scenario in which the loci were in LD and only SNPs were modeled. As a consequence the true value of *π *was unknown. In both scenarios, loci were biallelic with initial allele frequency of 0.5, and QTL effects were sampled either from a standard normal or from a gamma with shape 0.4 and scale 1.66 as in [1]. Figure 1 depicts the cumulative distribution functions of these two distributions to illustrate the different effect sizes simulated. The sampled QTL effects were standardized before training to exhibit the additive-genetic variance calculated from a specified heritability and a residual variance of 1. Trait phenotypes were simulated by adding residual effects sampled from a standard normal to the sum of the genotypic values. Simulations were varied with different numbers of QTL and training data sizes, which was either 1,000 or 4,000 individuals. The MCMC algorithms were run for 50,000 iterations with a burn-in of 20,000 iterations. A higher number of iterations did not change the results.

In the ideal simulations, a total of 2,000 loci were simulated as if they were all located on different chromosomes to ensure linkage equilibrium. The number of QTL among those loci was 10, 200, or 1,000 and trait heritability was 0.5. The realistic simulations started with a base population of 1,500 individuals that were randomly mated over 1,000 generations to generate LD from mutations and drift. Individuals of generation 1,000 were used as founders of a real pedigree from the North American Holstein population, which included 7,094 bulls used in the real data analysis. This simulated LD similar to that in real livestock populations [9]. Individuals from the last generation of pedigree individuals were parents of the training individuals, with each parent represented once. The simulated genome consisted of a single chromosome of length 1 M that had 4,000 evenly-spaced SNPs and either 10, 20, or 40 QTL that were randomly distributed on the chromosome. The mutation rate was 2.5•10^-5 ^for both SNPs and QTL, which is larger than estimates of actual mutation rates to ensure that a sufficient number of loci was segregating after 1,000 generations of random mating; it can be shown that mutation rate has only a small effect on LD in this simulation using the formula derived by [18]. Recombinations were modeled according to a binomial map function, where the maximum number of uniformly and independently distributed crossovers on a chromosome of 1 M was 4 [19], i.e., assuming interference. The proportion of segregating loci after 1,000 generations of random mating was 0.98, hence the number of segregating QTL in the scenarios with 10, 20 and 40 QTL was 9.8, 19.6 and 39.2 on average, respectively. To select 2,000 SNPs for training and validation, the chromosome was first divided into 2,000 evenly-spaced bins and then one SNP with minor allele frequency greater than 0.05 was randomly selected in each bin. The heritability was varied with the values 0.03, 0.2 and 0.9 to modify the size of QTL effects. All simulations were repeated 24 times.

## Real data analyses

Data from North American Holstein bulls were used to gain information about the number of QTL affecting quantitative traits in real populations and to compare the different Bayesian methods with respect to GEBV accuracy that results mainly from LD information.

### Genotyped bulls

The data set consisted of 7,094 progeny tested North American Holstein bulls that were genotyped for the Illumina Bovine50K array, excluding bulls that had more than 5% missing genotypes. De-regressed breeding values obtained from the official genetic evaluation of the USDA in August 2009 were used as trait phenotypes and were available for the quantitative traits milk yield, fat yield, protein yield and somatic cell score. The de-regressed proofs of the bulls used had a reliability greater than 0.7 and the square root of the reliability was used to weight residual effects [20]. The average reliability of milk, fat and protein yield was 0.89 and that of somatic cell score 0.81. Furthermore, a pedigree, containing the bulls in cross-validation and their ancestors born after 1950, was available to model polygenic effects and to quantify additive-genetic relationships between training and validation bulls.

### SNP data

SNPs were selected for the analyses based on minor allele frequency (> 3%), proportion of missing genotypes (< 5%), proportion of mismatches between homozygous genotypes of sire and offspring (< 5%) and Hardy-Weinberg equilibrium (*p *< 10^-10^). The total number of SNPs in the analyses was 40,764.

### Training and validation data sets

Bulls born between 1995 and 2004 were used for training, whereas 113 bulls born before 1995 and with additive-genetic relationships to the training bulls smaller than 0.1 were used for validation. The reason for generating this cross-validation scenario was that LD rather than additive-genetic relationships was to determine the accuracy of GEBVs. The contribution of LD information to the estimates of SNP effects is sensitive to the size of the training data set, and thus 1,000, 4,000 and 6,500 training bulls were randomly selected from the bulls born between 1995 and 2004.

The MCMC algorithms were run for 200,000 iterations with a burn-in of 150,000 iterations for 1,000 training bulls, 100,000 iterations with a burn-in of 50,000 iterations for 4,000 training bulls, and 50,000 iterations with a burn-in of 20,000 iterations for 6,500 training bulls. These numbers of iterations were sufficient in that a higher number did not change the results. Posterior distributions were visually inspected for convergence. In addition to the GEBVs obtained by the Bayesian model averaging methods, breeding values for the validation bulls were estimated using an animal model with the numerator-relationship matrix [21,22], which provided standard pedigree-based BLUP-EBVs (P-BLUP) to quantify the genetic-relationship information from the pedigree. An animal model with a genomic relationship matrix [13] was used to obtain GEBVs (G-BLUP), which is equivalent to ridge-regression.

### Evaluation criteria

Estimates of *π *were studied as , where *K *is the number of loci used in the statistical analysis. This represents the posterior mean of the number of loci fitted in each iteration of the MCMC algorithm (*N*_SNP_), which is more practical than *π *for comparisons of scenarios that differ in the number of simulated QTL. The reason is that the true value of *π *is usually unknown unless QTL are among the loci in the model. The accuracy of GEBVs was estimated by correlation between GEBVs and de-regressed proofs divided by the average accuracy of de-regressed proofs of the validation bulls. The GEBV of validation bull *i *was calculated as

where *z_ik _*is the genotype score (0, 1, or 2) for bull *i *at SNP *k *and  is the posterior mean of the effect at that locus. The EBVs from P-BLUP and G-BLUP were obtained from solutions of the animal model.

## Results

### Ideal scenario

Table 2 depicts the posterior number of SNPs fitted in the model (*N*_SNP_) estimated by BayesC*π *and BayesD*π *starting with *π *= 0.5 according to the number of training individuals, number of QTL (*N*_QTL_) and distribution of QTL effects, which all had a considerable effect on the results. A sufficiently large set of training data would include in the model only QTL and no spurious SNPs. With normal distributed QTL effects, BayesC*π *was more accurate in this regard than BayesD*π*, especially as training data size increased. BayesD*π *fitted substantially more loci than *N*_QTL _in the scenarios with 10 and 200 QTL; in addition, *N*_SNP _did not approach *N*_QTL _as training data size increased in the scenario with 1,000 QTL. With gamma distributed QTL effects, *N*_SNP _was always lower than *N*_QTL _with BayesC*π*; BayesD*π*, in contrast, overestimated *N*_QTL _when 10 and 200 QTL were simulated, but underestimated it for 1,000 QTL. Starting with *π *= 0.8 or 0.95 hardly changed *N*_SNP _from BayesC*π *(results not shown); the only notable change was obtained for normal distributed QTL effects and *N*_QTL _= 1,000, where *N*_SNP _increased with training data size from 640 to 957.

**Table 2:** Posterior mean of (1-*π*) multiplied by *K *= 2,000 loci used in the analyses (se) according to the Bayesian method, number of QTL, distribution of QTL effects and training data size.

		QTL effect distribution and training data size			
		Gamma		Normal	
		
Method	No. of QTL	1,000	4,000	1,000	4,000
BayesC*π*	10	7 (1)	7 (0.8)	13 (0.9)	12 (0.8)
	200	69 (5)	86 (3)	236 (13)	204 (3)
	1,000	312 (40)	315 (8)	1,230 (91)	1,007 (19)
BayesD*π*	10	165 (11)	59 (3)	229 (9)	81 (4)
	200	645 (22)	343 (7)	952 (24)	564 (6)
	1,000	984 (39)	747 (10)	1,169 (36)	1,227 (12)

The starting value for *π *was 0.5. Results are based on 24 replicates of the ideal simulation in which all loci were in linkage equilibrium and both SNPs and QTL were modeled

### Realistic scenario

Table 3 shows *N*_SNP _estimated by BayesD*π *for *h*^2 ^= 0.9. Although *N*_SNP _declined with decreasing *N*_QTL_, *N*_SNP _overestimated *N*_QTL _considerably, and the training data size did not have an effect on *N*_SNP_. The overestimation was even higher for heritabilities of 0.03 and 0.2 (not shown), but *N*_SNP _decreased somewhat with increasing training data size. BayesC*π *with a starting value of *π *= 0.5 (Table 4) overestimated *N*_QTL _less than BayesD*π *for *h*^2 ^= 0.9, and significant trends were obtained for *N*_SNP _with increasing training data size, which depended on the distribution of QTL effects, *N*_QTL_, and *h*^2^. For *h*^2 ^= 0.9, *N*_SNP _increased with training data size and the overestimation of *N*_QTL _decreased with *N*_QTL_. For *h*^2 ^= 0.2, in contrast, the overestimation was higher with 1,000 training bulls, and *N*_SNP _decreased significantly with training data size. For *h*^2 ^= 0.03, *N*_SNP _was generally high, and decreased with training data size except for 20 and 40 QTL with normally distributed effects. However, the trend with training data size for *h*^2 ^= 0.03 was smaller than for the other two *h*^2 ^values relative to the high *N*_SNP _with 1,000 training individuals. Starting with *π *values of 0.8 and 0.95 (results not shown) did not change results for *h*^2 ^= 0.9, but decreased the decay of *N*_SNP _with training data size for *h*^2 ^= 0.2, because estimates for *N*_SNP _were smaller with 1,000 training individuals. The latter was also observed for *h*^2 ^= 0.03 along with a decreasing trend for *N*_SNP_.

**Table 3:** Posterior mean of (1-*π*) multiplied by *K *= 2,000 SNPs used in the analyses (se) obtained by BayesD*π *according to the number of QTL, distribution of QTL effects and training data size.

	QTL effect distribution and training data size			
	Gamma		Normal	
	
No. of QTL	1,000	4,000	1,000	4,000
10	243 (14)	253 (14)	375 (23)	395 (21)
20	278 (24)	293 (25)	546 (31)	538 (29)
40	461 (30)	465 (26)	779 (31)	771 (19)

Results are based on 24 replicates of the realistic simulation in which heritability was 0.9, loci were in linkage disequilibrium, and only SNPs were modeled

**Table 4:** Posterior mean of (1-*π*) multiplied by *K *= 2,000 SNPs used in the analyses (se) obtained by BayesC*π *according to the heritability (*h*^2^), number of QTL, distribution of QTL effects and training data size.

		QTL effect distribution and training data size			
		Gamma		Normal	
		
*h*^2^	No. of QTL	1,000	4,000	1,000	4,000
0.9	10	52 (5)	99 (9)	73 (5)	147 (7)
	20	65 (6)	127 (11)	112 (7)	210 (10)
	40	115 (11)	198 (13)	202 (19)	343 (17)

0.2	10	421 (137)	37 (5)	532 (115)	54 (5)
	20	654 (140)	62 (8)	917 (131)	133 (35)
	40	1006 (97)	174 (57)	1178 (42)	434 (109)

0.03	10	1083 (80)	933 (130)	1045 (59)	1081 (108)
	20	1162 (69)	1103 (58)	1035 (50)	1099 (62)
	40	1043 (83)	1206 (42)	1149 (54)	1331 (39)

Starting value for *π *was 0.5. Results are based on 24 replicates of the realistic simulation in which loci were in linkage disequilibrium and only SNPs were modeled

### Real data analyses

Additive-genetic relationships between training and validation bulls were small: No validation bull had an additive-genetic relationship to a training bull exceeding 0.092. The distribution of the maximum additive-genetic relationships between training and validation bulls had a lower quartile, median, and upper quartile of 0.016, 0.05 and 0.07, respectively. The main cause of the low additive-genetic relationships was a separation of about three generations between the bulls of both data sets, because 90% of the validation bulls were born before 1975. Table 5 shows accuracies of P-BLUP, G-BLUP, and the Bayesian model averaging methods according to the quantitative trait and training data size. The accuracies of P-BLUP for fat and protein yield as well as somatic cell score were close to zero as expected, but the accuracy for milk yield was unexpectedly high with 0.15 and 0.24 for 1,000 and 4,000 training individuals, respectively.

**Table 5:** GEBV accuracy of 113 Holstein Friesian bulls born between 1953 and 1994 according to the Bayesian method, quantitative trait and number of Holstein Friesian bulls born between 1995 and 2004 used for training.

Trait	Training data size	P-BLUP	G-BLUP	BayesA	BayesB, *π *= 0.99	BayesC*π*	BayesD*π*
Milk yield	1,000	0.15	0.38	0.39	0.22	0.35	0.38
	4,000	0.24	0.46	0.46	0.41	0.43	0.46
	6,500	0.10	0.48	0.48	0.40	0.43	0.47
	
Fat yield	1,000	-0.05	0.41	0.48	0.51	0.48	0.47
	4,000	0.04	0.49	0.54	0.55	0.58	0.56
	6,500	-0.15	0.51	0.56	0.52	0.54	0.57
	
Protein yield	1,000	0.02	0.13	0.14	0.05	0.14	0.13
	4,000	0.03	0.17	0.17	0.13	0.17	0.16
	6,500	-0.02	0.21	0.22	0.17	0.21	0.20
	
Somatic cell score	1,000	0.03	0.04	0.06	0.06	0.06	0.05
	4,000	-0.11	0.14	0.18	0.12	0.15	0.16
	6,500	-0.04	0.17	0.17	0.12	0.14	0.14

Starting value of *π *in BayesC*π *was 0.5.

standard error,

Accuracies of GEBVs were similar for the different methods with the following exceptions: BayesB with *π *= 0.99 had the lowest accuracies for all traits but fat yield, and G-BLUP had the lowest accuracies for fat yield. Furthermore, the accuracies for milk yield obtained by BayesC*π *tended to be lower than for G-BLUP, BayesA and BayesD*π*. In general, BayesA tended to give the highest accuracies for all traits except for fat yield. The accuracies of BayesC*π *did not differ depending on the starting values for *π *(results only shown for starting *π *= 0.5).

The accuracy of GEBVs improved markedly with training data size for milk yield, fat yield and somatic cell score from 1,000 to 4,000 bulls, but improved only slightly or reduced from 4,000 to 6,500 bulls. The increase in accuracy with training data size for protein yield was less than for the other traits from 1,000 to 4,000 bulls, but tended to be more from 4,000 to 6,500 bulls. Somatic cell score had the highest relative increase in accuracy of all traits because accuracies were lowest for 1,000 training bulls. Interestingly, G-BLUP had the lowest accuracy for somatic cell score with 1,000 training bulls, but the increase was largest such that the accuracy for 6,500 bulls was as high as for BayesA.

The posterior distributions for *N*_SNP _(not shown) were unimodal, symmetric, and standard deviations decreased notably with increasing training data size as in Table 6. Exceptions were the posterior distributions for protein yield and somatic cell score of BayesC*π *with 1,000 training bulls, which were bimodal and rather flat. Although the accuracies of BayesC*π *and BayesD*π *were very similar, they fitted very different numbers of SNPs (Table 6). As in the realistic simulations, *N*_SNP _from BayesD*π *was insensitive to training data sizes for all traits, whereas BayesC*π *showed clear trends with training data size that differed across traits; *N*_SNP _was comparatively low for milk and fat yield and increased with training data size, and estimates were very similar for the different starting values of *π*, *π*_0_. *N*_SNP _always decreased with training data size for protein yield, but estimates increased for all training data sizes as *π*_0 _decreased. For somatic cell score, however, the trends changed depending on *π*_0_; *N*_SNP _increased with training data size for *π*_0 _= 0.95, but decreased with lower *π*_0 _values.

**Table 6:** Posterior mean () and standard deviation () of (1-*π*) obtained by BayesC*π *(Starting value of *π *was 0.5) and BayesD*π *multiplied by *K *= 40,764 SNPs used in the analyses, and average number of SNPs () fitted by BayesB with *π *= 0.99 and standard error (*se*) according to the quantitative trait and the number of Holstein Friesian bulls used for training

		BayesB, *π *= 0.99	BayesC*π*		BayesD*π*	
		
Trait	Training data size	(*se*)				
Milk yield	1,000	402 (1.5)	2,119	545	13,982	1,793
	4,000	436 (1.6)	2,315	398	13,329	896
	6,500	518 (1.6)	2,555	326	14,768	750
	
Fat yield	1,000	401 (1.1)	562	201	13,533	1,752
	4,000	441 (1.3)	1,488	210	13,513	895
	6,500	504 (1.3)	2,058	229	13,703	631
	
Protein yield	1,000	403 (0.9)	10,986	3,970	14,430	2,201
	4,000	438 (1.1)	9,500	1,756	13,512	774
	6,500	514 (1.1)	5,503	970	14,496	694
	
Somatic cell score	1,000	398 (1.2)	5,644	3,105	12,962	1,948
	4,000	428 (1.3)	3,624	1,043	13,941	954
	6,500	466 (1.3)	2,723	508	13,464	741

## Discussion

Two Bayesian model averaging methods that address the statistical drawbacks of BayesA and BayesB were developed for genomic prediction. These two models were termed BayesC*π *and BayesD*π *to emphasize that the prior probability *π *that a SNP has zero effect was treated as an unknown. The objectives of this study were to evaluate the ability of these methods to make inferences about the number of QTL (*N*_QTL_) of a quantitative trait by simulated and real data, and to compare accuracies of GEBVs from these new methods compared to existing methods.

### Simulations

In ideal simulations, all loci were in linkage equilibrium and both SNPs and QTL were modeled. BayesC*π *was able to distinguish the QTL that had non-zero effects from the SNPs that had zero effects as training data size increased and when QTL effects were normally distributed. In contrast, when QTL effects were gamma distributed many QTL remained undetected. This may have been because the gamma distribution generated fewer large effects and more small effects than the normal (Figure 1). Further, the prior of SNP effects in BayesC*π *given the common effects variance was normal and not gamma; a gamma prior may produce better results and should be investigated in a subsequent study. In conclusion, even in this ideal case the estimate of  obtained from BayesC*π *is a poor indicator for *N*_QTL_, unless the QTL distribution is normal. BayesD*π *was insensitive to *N*_QTL _and inappropriate to estimate *N*_QTL_.

In realistic simulations, SNPs and QTL were in LD and only the SNP genotypes were known. As expected, BayesC*π *fitted more SNPs than there were QTL, because every QTL was in LD with several SNPs. However, the number of SNPs fitted per QTL depended on both training data size and effect size of a QTL, which was varied here by the distribution of QTL effects, *h*^2 ^and *N*_QTL_; the size of simulated QTL effects increased with *h*^2 ^and decreased with *N*_QTL_. If QTL effects were generally large and easy to detect (Table 4, *h*^2 ^= 0.9), *N*_SNP _was small with 1,000 training individuals and increased with training data size. In addition, the larger a QTL effect, the more SNPs were fitted per QTL (Table 4, *h*^2 ^= 0.9, 10 vs. 20 *N*_QTL_). The cause for these findings may be that SNPs in low LD with the QTL were more likely to be fitted as either QTL effect size or training data size increased. The increase in *N*_SNP _with training data size could also have been the result of detecting QTL with smaller effects. If QTL effects were smaller and less easy to detect (Table 4, *h*^2 ^= 0.2), *N*_SNP _was larger with 1,000 training individuals, which may be explained by false positive SNPs in the model, because the power of detection was likely to be low. In contrast to *h*^2 ^= 0.9, *N*_SNP _decreased substantially with training data size. However, the fact that *N*_SNP _increased with training data size for *h*^2 ^= 0.03, normally distributed QTL effects, and a starting value of *π *= 0.5 ( small) points to another explanation why many SNPs were fitted with small QTL effect size or small training data size: a higher number of SNPs explains differences between training individuals better than a smaller number, and thus more SNPs were required to explain those differences as training data size increased. In conclusion, BayesC*π *overestimates *N*_QTL_, the extent depending on the size of QTL effects, which makes inference difficult. However, information about *N*_QTL _can be gained by analyzing the trend of *N*_SNP _with training data size, and starting with different *π *values. Furthermore, as SNP density increases in the future, overestimation of *N*_QTL _is expected to be smaller, because LD between SNPs and QTL will be higher such that fewer SNPs are modeled per QTL. Sufficiently high SNP density guarantees near perfect LD between at least one SNPs and each QTL in which case the scenario of the ideal simulations will be approached.

### Real data analysis

#### Number of QTL and size of QTL effects

In agreement with the realistic simulations, estimates of *π *from BayesD*π *were insensitive to both trait and training data size (Table 6). BayesC*π*, in contrast, showed clear differences for both: *N*_SNP _increased with training data size for milk and fat yield, and decreased for protein yield and somatic cell score. Thus, milk and fat yield may have more QTL with large effects than protein yield and somatic cell score, which can be derived from the trends of *N*_SNP _in the realistic simulations. This is also supported by the accuracies of GEBVs where milk and fat yield had a higher accuracy than protein yield and somatic cell score. Furthermore, fat yield may have more QTL with large effect than milk yield, because both the increase of *N*_SNP _from 1,000 to 4,000 training bulls and accuracy of GEBVs was higher for fat yield.

The number of SNPs in the model estimated by BayesC*π *may primarily result from the QTL with the largest effects, assuming that QTL with small effects were not detectable. The rather low accuracies of GEBVs and especially the low increase in accuracy from 4,000 to 6,500 bulls may also point to this conclusion, because many more training individuals seem to be necessary to estimate small QTL effects. Another reason may be that LD between SNPs and QTL was still too low, but this will change as SNP density increases; QTL with large effects will be estimated with fewer SNPs and additional QTL with smaller effect will be detected.

As mentioned earlier, a possible overestimation of *N*_QTL _results from the fact that several SNPs are in LD with a QTL, where each of these SNPs explains a part of the QTL effect. These SNPs are likely to surround the QTL on the chromosome, and thus *N*_QTL _can be estimated more precisely by calculating the variance of GEBVs explained by the effects of all SNPs in a specified chromosomal region. This can be done by defining a window containing a certain number of consecutive SNPs that are used to calculate this variance. By sliding the window over the chromosome and observing peaks that are higher than for single SNPs, *N*_QTL _may be inferred better. This can be done with all methods that estimate SNP effects.

#### Comparison of the accuracy of GEBVs

North American Holstein bulls were partitioned into training and validation data sets such that bulls of both data sets were as unrelated as possible. As a result, the contribution of additive-genetic relationships to the accuracy of GEBVs was negligible for fat yield, protein yield and somatic cell score as demonstrated by the low accuracy of P-BLUP. However, that accuracy was unexpectedly high for milk yield, which might be an artifact of previous selection for milk yield because genotypes in the validation data set were only available from selected parents. Accuracies of GEBVs were similar for the different methods, and no one outperformed all the others across all traits or training data sizes. Nevertheless, BayesA performed remarkably well for this SNP density despite the statistical drawback of BayesA as described by [11]. However, as demonstrated in [11], it is important that the degrees of freedom used for the scaled inverse chi-square prior of the locus-specific variances express little prior belief. BayesA always fits all SNPs, hence the shrinkage of SNP effects results completely from the locus-specific variances, and, in contrast to the other methods, SNP effects are not fully shrunk to zero. Thus, even SNPs that truly have zero effects are expected to have small estimated effects adding noise to the GEBVs. This applies also to G-BLUP, which is equivalent to ridge-regression fitting all SNPs with equal variance. This did not seem to affect the accuracy of GEBVs here, but in the simulations of [1] BayesB performed better than BayesA and ridge-regression. The explanation may be that the traits analyzed here are determined by many more QTL than in those simulations. Thus, BayesA may be inferior to BayesC*π *and BayesD*π *for traits that are determined by only a few QTL and when many more SNPs effects are modeled as SNP density increases. Applying BayesA to the data sets of the realistic simulations with only 10 QTL confirmed its inferiority to BayesC*π *and BayesD*π*.

Treating *π *as known with a high value as in BayesB may be a poor choice. This agrees with Daetwyler et al. [23] who reported that G-BLUP outperformed BayesC with a fixed *π *when the number of simulated QTL was large. This can be explained partly by the fact that [23] considered the GEBV accuracy of the offspring of training individuals, meaning that genetic-relationships were important; these were captured better by the SNPs, when more SNPs were fitted as in G-BLUP [7]. Note further that BayesC with *π *= 0 is similar to G-BLUP. Consider ridge-regression as the equivalent model of G-BLUP to see this similarity. Both methods are equivalent either 1) if the single effect variance of BayesC is treated as known, 2) if *ν_a _*is very large and  equals to the single effect variance of ridge regression, or 3) if the single effect variance of ridge regression is treated as unknown with own scaled inverse chi-square prior. Thus the lower accuracy for BayesC in that study results most likely from treating *π *as known. Another reason may be that the scale parameter of the inverse chi-square prior for the common effect variance in [23] did not depend on the additive-genetic variance nor on the fixed *π *value as proposed by [1].

The finding that BayesC*π *and BayesD*π *give similar accuracies but different *π *values reveals that the two methods have different mechanisms for shrinking SNP effects. BayesD*π *primarily used the locus-specific variances, whereas BayesC*π *was only able to vary the shrinkage at different SNPs by using *δ_k_*; if a SNP is not fitted to the model the effect is shrunk completely to zero, otherwise they are all shrunk using the same ratio of residual to common effect variance. In principle, BayesD*π *is expected to be more flexible in shrinking SNP effects because it could use both locus-specific variances and *δ_k _*for this purpose. The poor mixing of *π *in BayesD*π *indicates that locus-specific variances dominated over *δ_k_*, which may explain why *π *is not an indicator for *N*_QTL_.

#### Effect of training data size on Bayesian model averaging

**Figure 2:**
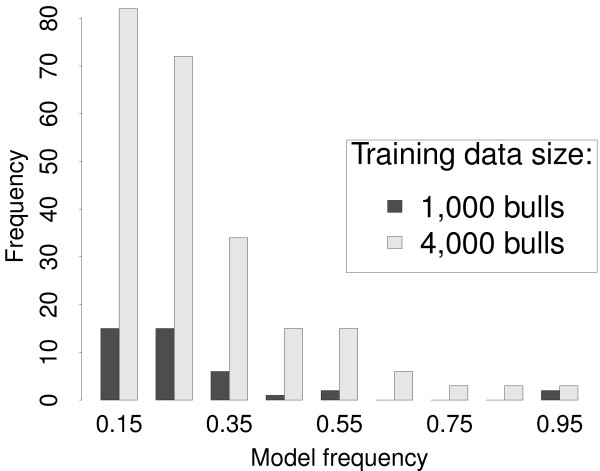
**Histogram of model frequencies > 0.1 obtained by BayesB with *π *= 0.99 using 1,000 and 4,000 training bulls**.

Another insight into the mechanisms of Bayesian model averaging comes from the large increase in accuracy of GEBVs with training data size obtained by BayesB for milk yield. The parameter *π *was treated as known with value 0.99 resulting in about 400 SNPs fitted in each iteration of the MCMC algorithm for both 1,000 and 4,000 training bulls (Table 6). This indicates that *π *is a strong prior for *δ_k _*= 0. Therefore, setting *π *= 0.99 is analogous to searching for models that fit about 400 SNPs in each iteration of the algorithm and to average them. These models change from one iteration to another as some SNPs are removed from the model, while others are included. This interchange of SNPs, however, is expected to be more frequent with a small training data size, because the power to detect significant SNPs is low. On the other hand, if the training data size is large, fewer SNPs are interchanged less often so that models differ less from one iteration to the other. This becomes most apparent in the increasing number of SNPs having moderate to high model frequency as training data size increased from 1,000 to 4,000 bulls as shown in Figure 2. The implication is that the effects of those SNPs were less shrunk with larger training data size, whereas effects of all other SNPs were shrunk more.

#### Comparison of GEBV accuracy with other studies

Accuracies of GEBVs reported by [24] and [2] for the North American and Australian Holstein populations, respectively, are not comparable to the accuracies found here. Accuracies for the milk production traits were higher in those studies, because validation bulls were closely related to those comprising the training data as demonstrated by [9]. In that study, accuracy of GEBVs due to LD was estimated from 1,048 and 2,096 German Holstein bulls using BayesB with *π *= 0.99. Most of those bulls were born between 1998 and 2004, and 60% were offspring of North American Holstein bulls revealing the high genetic relationships between the German and the North American Holstein population. The strategy used to estimate the accuracy due to LD was a regression approach based on pairs of training and validation data sets with different additive-genetic relationships between the bulls of both data sets. That strategy is very time-consuming when several methods must be compared, and therefore a different approach was chosen here. GEBV accuracies obtained by BayesB compared to those in [9] were similar for milk yield, comparable for fat yield when the training data size was greater than 1,000, but lower for protein yield and somatic cell score. The increase of accuracy with training data size tended to be higher in [9]. Moreover, in contrast to the present study, G-BLUP was inferior in [9]. The difficulties in comparing the accuracies found here to those in [9], apart from the standard errors, is that there might be genotype-environment interactions, because the environment in which the daughters of the bulls born before 1975 have been tested might be different from the environment of the last decade relevant to the daughters of the training bulls. In addition, selection and genetic drift may have changed the LD structure in the population so that the accuracies of this study may not represent the GEBV accuracies due to LD in the current population.

#### Computing time

Computing time, which may become more important as SNP density increases, is an advantage of BayesC*π*, because its Gibbs algorithm is faster than the Metropolis-Hastings algorithm of the other methods. The reason is that the MH step for sampling the locus-specific variances in this implementation of BayesA, BayesB and BayesD*π *is repeated in each iteration to improve mixing; the Gibbs step for fitting a SNP in BayesC*π *is only performed once. Furthermore, computing time depends largely on the number of SNPs fitted in each iteration, because the following two computation steps are the most demanding ones in the algorithm: The phenotypes have to be unadjusted for the genotypic effects of a SNP if that SNP was fitted in the previous iteration; similarly, if a new SNP effect was sampled in the current iteration, the phenotypes have to be adjusted for the genotypic effects of that SNP. BayesC*π *was more sensitive to both the genetic architecture of a trait and training data size than BayesD*π*, and thus computing time was shorter for BayesC*π*. In this implementation, BayesA always had the longest computing time because all SNPs were fitted. For example, using 1,000 training bulls for milk yield and a 2.4 GHz *AMD 280 Opteron *processor, computing time for 100,000 iterations was 10.3, 14.1, 18 and 21.3 hr for BayesC*π*, BayesB, BayesD*π *and BayesA, respectively.

## Conclusions

BayesC*π *and BayesD*π *that address the drawback of BayesA and BayesB regarding the impact of the prior hyperparameters on shrinkage of SNP effects and that treat as an unknown the prior probability *π *that a SNP has zero effect were developed for genomic prediction. Estimates of *π *from BayesC*π*, in contrast to those from BayesD*π*, are sensitive to training data size and SNP density, and provide information about the genetic architecture of a quantitative trait; the traits milk yield and fat yield measured in North American Holsteins have QTL with larger effects than protein yield and somatic cell score. The statistical drawback of BayesA and BayesB did not impair the GEBV accuracy that is mainly due to LD information. Accuracies of the alternative Bayesian methods were similar and none of them outperformed all others across all traits and training data sizes. Therefore the best method must be determined for each quantitative trait separately. In contrast to simulation studies, BayesA was a good model choice for genomic prediction in the North American Holstein population at this current SNP density. Treating *π *as known with a high value is not recommended as alternative methods such as BayesC*π *or BayesD*π *gave better accuracies. In general, computing time is shorter for BayesC*π *than for BayesD*π*, and longest for BayesA. Collectively, accounting for computing effort, uncertainty as to the number of QTL (which affects the GEBV accuracy of alternative methods), and fundamental interest in the number of QTL underlying quantitative traits, we believe that BayesC*π *has merit for routine applications.

## Competing interests

The authors declare that they have no competing interests.

## Authors' contributions

All authors contributed to the development of the statistical methods and to the program code of *GenSel *software. DH conducted the analyses and drafted the manuscript. All other authors contributed to the final manuscript, read and approved it.

## Supplementary Material

Additional file 1 ***GenSel *- user manual 2009**. Click here for file
