# Supplementary material for: Extension of the bayesian alphabet for genomic selection
Source: BMC Bioinformatics. 2011 May 23;12:186. doi: 10.1186/1471-2105-12-186 (PMC3144464; doi:10.1186/1471-2105-12-186)
Supplement: Additional file 1 — GenSel - user manual 2009. [file 1471-2105-12-186-S1.PDF]

GenSel – User Manual for a portfolio of Genomic Selection related Analyses

Rohan Fernando and Dorian Garrick

Iowa State University

Third Edition refers Version 2.14 May 2009

Second Edition refers Version 2.12 May 2009

First Edition refers Version 2.0 December 2008

Introduction

GenSel is a computer program principally written by Rohan Fernando in C++ that comprises a portfolio of alternative analyses relevant to Genomic Selection. It requires three input files; two of which comprise Genotypic and Phenotypic data; and a command file that specifies relevant analytical options.

GenSel was developed on Mac platforms using GCC 4.3.0. It requires libraries from GSL, MatVec, and Boost, and an installation of Gnuplot to visualize posteriors.

GenSel is available to ISU research collaborators in Genomic Selection but is not for public distribution. Its analytical options will be available via a web interface designed for beef cattle/bovine analyses. Those activities are supported by funding from USDA NRI 2008-03924 competitive grant.

This manual describes the broad family of analytical options that are available in GenSel and provides relevant details as to input file formats and outputs.

Direct any questions or comments by email to dorian@iastate.edu

Table of Contents

Introduction 1

Table of Contents 2

Running GenSel 3

Genotypic file format (ascii/text) 4

Phenotypic file format 5

Binary Genotype File 7

Output file naming conventions 8

Generic options 8

Bayesian Analyses 12

- BayesA 13
- BayesB 13
- BayesC 14
- BayesCPi 14
- BayesCCSub 15

Output options for Bayes Analyses 16

Pathogenic Datasets 18

Predicting Genomic Merit 19

Least Squares 20

Simulating Data for Analysis 21

Linkage Disequilibrium 22

Keyword Summary 23

Running GenSel

GenSel can be invoked from the terminal by typing

GenSel

which will result in the program reporting the version number.

The program is normally invoked with an argument defining the analytical options. Conventionally, this file has the suffix .inp. For example,

GenSel Example.inp

will invoke GenSel with the command options specified in the file Example.inp and the output files will begin with Example and end with various suffixes depending upon the options specified. Specific details about analytical options will follow in the corresponding sections for each analysis option. However, all options require specifying the filename for the genotypes and the file name for the corresponding phenotypes. Additional options can be used if desired in any analysis to limit the availability of genotypes to avoid having to create copies of subsets of the genotype file.

Genotypic file format (ascii/text)

The genotype file is entered as a space-delimited matrix of covariates, following a header line. After the header line, the first column is an alphanumeric representing the animal identifier and the following columns are covariates, typically scored for substitution effects as -10, 0 or 10 representing for example AA, AB and BB genotypes. Missing genotypes are not handled. In the event genotypes were missing on a few animals, one option is to replace the missing code with the average value, or the average value for that particular breed. Another option is to use separate software to predict haplotypes and infer the missing genotypes. The header line has a character description of the ID, such as animal, followed by an integer to represent a code for each column of genotypes. This code will be used in the output to identify a particular locus. We typically label the columns to represent their ordered genomic location. There are no limits on the number of animals contained in the genotypic file. The file therefore contains a matrix with one more row than the number of animals with genotypes and one more column than the number of covariates representing substitution effects. The rows and the columns can be arbitrarily ordered, other than the requirement that the first row is the header and the first column are the animal identifiers. Only those rows that correspond to animal identifiers represented in the phenotype file will be stored in memory and used in the analysis.

```
          Example_genotypic.data
Anim_ID    1   2    5  99
Donald   -10 10  -10   0
Duck     -10  0    0  10
Mickey     0  10 -10  10
Mouse      5   0 -10   0
James007 10 10     0 -10
```

Covariates may exhibit no variation (eg all values of -10) or may be identical to other covariates in the data file (eg two identical columns).

A genotype file representing 50k covariates will be around 200 megabytes per 1,000 animals or 1 gigabyte per 5,000 animals. Reading this file in ascii or text form is time consuming, so it is best to convert it to binary form using the gen2bin program (see later).

Phenotypic file format

The phenotypic file defines the relevant trait values for a single phenotypic trait along with describing the model equation, if any, to be used in the analysis of that trait. There is currently no support for multivariate analyses – each trait is recorded in a separate file. The phenotype file contains a header row, followed by a row for each animal with a trait measurement. Animals with missing values are not included in the phenotype file. Animals that are not in the genotype file may be included in the phenotype file, but will not be used in any analysis. Prior to any analysis, GenSel matches the animal identifiers in the genotypic and phenotypic file and stores only those that match their animal identifier in the two files. That is, the genotypic file can contain animals not in the phenotypic file and the phenotypic file can contain animals not in the genotypic file.

The minimum requirements for the phenotypic file are a header line that contains the descriptor for the animal identifier and a trait name. The remainder of the file would contain as many rows as there are animals with observations for the trait, each line containing the animal identifier in the same format as used in the genotypic file and the trait value, separated from the animal identifier by a space. The phenotypic file can optionally contain other information relevant to the analysis, as defined by descriptors in the header row. Two kinds of effects may be included, those relating to fixed effects in the form of class effects or covariates, and those relating to weighting factors to account for heterogeneous variance. These factors will be detailed below.

Any item identified by a name in the header row followed by a trailing # character will be ignored in the analysis. Any item named in the header and followed by a trailing $ character will be treated as a class variable and fitted with as many levels as there are unique alphanumerics in the data rows of the corresponding column label. Any item without a trailing # or $ will be treated as linear covariate. These fixed effects designators may be represented in any order in the phenotypic file. The particular keyword "rinverse" is reserved to designate the elements of a inverse of the diagonal matrix of residual variances. This element will be used in weighted analyses, for example when the trait observation is a progeny mean or estimated breeding value. A trailing # will result in an unweighted analysis. Fixed effects and weights in the phenotypic file will only be used in Bayesian and least squares analyses.

There are no restrictions on the use of paths or suffixes in the naming of phenotypic or genotypic files.

Example_phenotypic.data

```
Anim_ID IQ sex$ date_of_birth junk#
Duck 120 male 150 Republican
Bond 130 male 250 Democrat
Mouse 99 female 300 Republican
```

Example.inp

```
// Following lines are parameter-name parameter-value pairs
// anything to the right of the parameter value is ignored
markerFileName          ./Example_genotypic.data
phenotypeFileName       ./Users/Dorian/Example_phenotypic.data
//This is the minimum requirement for any analysis
// that is the genotypic & phenotypic file designators
```

AnotherExample.inp (This uses the binary genotype file)

```
// Following lines are parameter-name parameter-value pairs
// anything to the right of the parameter value is ignored
markerFileName          ./Example_genotypic.data.bin
phenotypeFileName       ./Users/Dorian/Example_phenotypic.data
//This is the minimum requirement for any analysis
// that is the genotypic & phenotypic file designators
```

Binary Genotype File

The genotype file is typically large with some 50,000 or more columns representing the SNP covariates, and may contain many thousands of animals, perhaps from a number of separate studies that may be suitable for pooled data analyses. The text form of the files is slow to read, and with thousands of animals the files themselves may be hundreds of megabytes in size. Converting these files to a binary form will halve the storage the requirement and results in minimal read time.

A genotype file with 50k SNP information on 10,000 animals will require about 1 gigabyte storage in binary form.

Separate software referred to as gen2bin can be run, with no arguments, from the terminal command prompt. The software will request the file name of the text version of the GenSel genotype file and will convert it to a binary file. The binary file will have the same name as the text file, except for a trailing .bin suffix.

GenSel will automatically read the binary rather than the text version of the genotype file if the markerFileName in the .inp file is modified by adding the .bin suffix.

For example the following line would read the binary form of the genotype file.

markerFileName Example_genotypic.data.bin

Output file naming conventions

Running GenSel will always create an output file such as Example.outn where n is a unique file designator, typically the digit 1 unless Example.out1 already exists in the default/working directory in which case the digit 1 will be progressively advanced (.out2, .out3 etc) until a unique designator is found for which an out file does not already exist. Any and all other output files from the same job will use the same digit identifier as the .out file. Accordingly, the user need only delete the file Example.out1 in order to ensure that successive analyses based on Example.inp overwrite the previous results.

Generic options

The genotype file is typically large (eg 1,000 animals with 50k genotypes may be 200 Mbytes) and many analyses may be of interest that include only subsets of the columns (eg particular SNP covariates). Accordingly, there are three mutually exclusive options for filtering the covariates in the genotypic file to include only a subset in any analysis. Filtering the rows (or animals) in the analysis is achieved by editing the corresponding phenotypic file that is usually of much smaller size than the genotypic file. Thus the genotypic file need never require copying and editing after its initial creation.

The first option to filter markers is by defining ranges of integer SNP identifiers that will be used to screen columns of covariates based on their integer names in the header of the genotypic file. The option is

```
includeRange        start:end
```

where start and end are integers such as 100:2005 which will result in only those markers with integer headings between 100 and 2005 being included in the analysis. This can be useful for example in selecting only those markers that are located on a particular chromosome, given that the naming convention for the covariates has been based on their chromosomal location. Multiple ranges can also be used, each range being separated by a decimal. For example

```
includeRange        100:500.9045:9432
```

will only use covariates with integer identifiers between 100 and 500 or 9045 and 9432. In the event that the includeRange option is not used, the option to specify markers for inclusion can be done by providing a filename, whereby that file contains no header and a simple list of integer covariate names, in any order. The keyword includeFileName is used to designate this option. For example

```
includeFileName     bfatn1.100markers
```

would result in only those covariates being used whose column headings in the genotypic file were included as a list in the file bfatn1.100markers. Note the file must contain only one column – that of the covariate names.

In the event that neither the includeRange or includeFileName options have been used, a filename may be designated to specify the covariate names to be deleted from the analysis. For example,

    excludeFileName   bfatn1.100markers

would result in only the covariates in the genotypic file that were not named as a list in the file bfatn1.100 markers to be included in any subsequent analysis.

All analyses will result in brief output to the screen to indicate the GenSel version number, the n suffix (outFileCount) that will be used in naming ouput files, followed by numbers of genotypic and phenotypic records that have been read and that have passed the filtering process for use in subsequent analyses.

```
doriang:/ dorian$ gensel test.inp
making matvec_trash directory
Genomic Selection Software: GenSel-2.11test
outFileCount 1
rows cols = 1721 41028
numberBytes to read = 141218376
 input model
 input phenotypic data
end of input phenotypic data

Number of records in genotype file:  1721
Number of records in phenotype file: 1692
Number of matching records:          1682
Number of marker loci in model:      41028
Number of fixed levels in model:     0
doriang:/ dorian$
```

File output is much more comprehensive. All jobs produce a file ending in .outn where n is the sequential outfile number, typically 1 unless this is a repeat job. The software version is then output, along with UTC time, the mean frequency of heterozygotes and numbers of genotypic and phenotypic observations and matching records. The information contained in the .inp file is then mirrored verbose in the .out file. Finally, relevant parameters as interpreted by the reading of the .inp file, along with any default values not set, are output. The nature of this information varies according to the analysis options used. It is useful for example to confirm whether the rinverse option was used. In Bayesian analyses, this file contains up to five different posterior means depending upon the options used. Finally the computing time is reported.

An example .out file

```
Genomic Selection Software:        GenSel-2.11test

UTC time: Thu May 14 13:29:46 2009

mean of 2pq = 0.370253
Number of records in genotype file:  1721
Number of records in phenotype file: 1692
Number of matching records:        1682
Number of marker loci in model:     41028
Number of fixed levels in model:    0

 Model Info:
   Model Term     Number of Levels
      Markers           41028

analysisType                  = Bayes
Marker Genotype File            = ../genotypes.test.bin
Input parameters:
*******************************************************
// Following lines are parameter-name parameter-value pairs
// anything to the right of the parameter value is ignored

markerFileName     ../genotypes.test.bin
phenotypeFileName   ../test.data

///markerSolFileName   ignore.mrkRes1
///includeFileName   incname

chainLength       410
burnin          10
analysisType      Bayes
bayesType           BayesCPi   //this jointly estimates pi from the data using BayesC
mcmcSamples       no      // This stores the samples for pi in a fie
plotPosteriors     yes      //This plots the posteriors (genvar, resvar, h2, pi - if mcmc yes)
probFixed        0.8
varGenotypic      .04
varResidual      .02
*******************************************************

Parameters used (including default values):
bayesType                        = BayesCPi
rinverse                         = no
```

```
chainLength                  = 410
burnin                       = 10
varGenotypic                 = 0.04
degreesFreedomEffectVar      = 4
varResidual                  = 0.02
nuRes                        = 10
probFixed                    = 0.8
mcmcSamples                  = yes
plotPosteriors               = yes
seed                         = 1234

 iter 0 number of loci in model 8015
 iter 100 number of loci in model 4999
 iter 200 number of loci in model 5025
 iter 300 number of loci in model 5248
 iter 400 number of loci in model 5237

 Posterior Mean of Residual Variance  = 0.0497556
 Posterior Mean of Genetic  Variance  = 0.0321692
 Estimated Total Variance             = 0.0819247
 Proportion of Variance a/c Markers   = 0.392667
 Posterior Mean of Pi                 = 0.86874
Computing Time in seconds: 89
```

End of example .out file

Bayesian Analyses

The principal analysis options for which GenSel was developed were to fit Markov Chain Monte Carlo (MCMC) methods for Bayesian analysis of high-density SNP data, the process known as "training" and the subsequent procedure referred to as "prediction" whereby the genomic merit is computed from SNP covariates alone. A number of alternative procedures for training have been developed, using Bayesian and Least Squares methodology. The nature of the output, and analytical options vary according to each procedure, described below.

The analytical options begin with a keyword known as analysisType. There are five analysis options. The most commonly-used option is

analysisType Bayes

while other options are Predict, StepWise, generateData and LD. The Bayes option is the most complex, with a number of suboptions. First is the bayesType keyword that identifies the broad category of the method among five options. These are `BayesA, BayesB, BayesC, BayesCPi, and BayesCCSub.`

The Bayes options have some generic parameters that apply to all types. Output options are described in a later section. The analysis options are given below. These include the chainLength or total number of realizations in the Markov chain. This number includes the pre- and post-burnin iterations. The burnin parameter defines how many of the chains will be ignored in summarizing the posterior distributions at the end of analyses. The prior genetic and residual variances are defined using the keywords varGenotypic and varResidual. The keyword seed defines the starting seed for the random number generator used in MCMC sampling. Two runs with the same seed will give identical results. The default seed is 1234. A user-defined seed can be integer or real. The keyword nuRes defines the degrees of freedom associated with the prior for the residual variance. The parameter degreesFreedomEffectVar defines the degrees of freedom for the prior for the covariates. The parameter probFixed defines the prior for pi. That is, a fraction 1-pi of the covariates will be fitted in each realization of the fitted Markov Chain. The number of Metropolis-Hastings iterations used in determining the variance for each covariate in BayesA and BayesB methods is defined by the keyword numMHIter. The default is 10 and increasing this number will proportionately increase run time. The order of any keywords, one per line, is completely arbitrary. Parameters associated with keywords for the Bayes options have default values so do not all need to be specified.

BayesA

The BayesA option invokes the BayesB method with pi=0, effectively fitting every covariate in every model in the chain. In this option the probFixed variable is ignored. The BayesA and BayesB methods fit a different variance for every covariate in the model. In the BayesA option the output model frequency for each covariate will be uninformative as they will all be unity.

Bayes A example (excluding marker and phenotype file definitions)

```
chainLength     41000    //this is total number of iterations
burnin          1000     //this many cycles are ignored
numMHIter       10       //This is for BayesB Metropolis Hastings
analysisType    Bayes    //Bayes, StepWise, Predict, generateData or LD
bayesType       BayesA   //BayesA, BayesB, BayesC, BayesCPi, BayesCCSub
varGenotypic    .00063
varResidual     .0017    //This should be same as scaleRes (round 1)
nuRes           10       // This is df for residual
```

BayesB

The BayesB option has identical requirements to BayesA, except that the parameter file should include a probFixed keyword to define the pi parameter, entered as a decimal between 0, and 1.

```
chainLength     41000    //this is total number of iterations
burnin          1000     //this many cycles are ignored
numMHIter       10       //This is for BayesB Metropolis Hastings
analysisType    Bayes    //Bayes, StepWise, Predict, generateData or LD
bayesType       BayesB   //BayesA, BayesB, BayesC, BayesCPi, BayesCCSub
probFixed       0.95
varGenotypic    .00063
varResidual     .0017    //This should be same as scaleRes (round 1)
```

BayesC

The BayesC method is preferred to BayesB in the sense that it is less sensitive to the varGenotypic prior and is a useful option to fit as a first analysis in order to estimate varGenotypic and varResidual priors. The priors for both parameters will be overwhelmed by the information contained in the data, whereas BayesB only achieves that for the varResidual parameter.

BayesC example (excluding marker and phenotype file definitions)

```
chainLength     41000     //this is total number of iterations
burnin          1000      //this many cycles are ignored
analysisType    Bayes     //Bayes, StepWise, Predict, generateData or LD
bayesType       BayesC    //BayesA, BayesB, BayesC, BayesCPi, BayesCCSub
probFixed       0.95
varGenotypic    .00063
varResidual     .0017     //This should be same as scaleRes (round 1)
```

BayesCPi

The parameter pi can be jointly estimated from the data using BayesCPi. In that case the BayesType is set to BayesCPi. The keyword probFixed is used to define the prior.

BayesCPi example (excluding marker and phenotype file definitions)

```
chainLength     41000     //this is total number of iterations
burnin          1000      //this many cycles are ignored
numMHIter       10        //This is for BayesB Metropolis Hastings
analysisType    Bayes     //Bayes, StepWise, Predict, generateData or LD
bayesType       BayesCPi //BayesA, BayesB, BayesC, BayesCPi, BayesCCSub
probFixed       0.5       // Starting value for estimating the posterior of pi
varGenotypic    .00063
varResidual     .0017     //This should be same as scaleRes (round 1)
```

The posterior for pi tends to be very flat except in the close neighborhood of the real solution. Accordingly, BayesCPi can take a long time to converge if a bad starting value is chosen, many markers are available and the trait is very polygenic. The estimates tend to be biased downwards. The procedure is very effective when the true pi is small (eg less than 100 QTL in simulated analyses with 50k markers). The posterior distribution (see output options) should be examined when this method is used, and the chainLength may need to be increased in relation to BayesC.

BayesCCSub

In circumstances whereby a subset of markers are of interest, one may be interested in fitting BayesC to identify the covariates with highest model frequency and then using these in subsequent BayesC analysis. This is achieved with the analysis BayesCCSub and the additional keywords subModelSize and numIterSubModel. For example, running a chain of 40,000 post burnin in BayesC then selecting the best 100 markers for a further 20,000 post burnin iterations would be achieved in the following example.

BayesCCSub example (excluding marker and phenotype file definitions)

```
chainLength      41000     //this is total number of iterations in the first chain
burnin           1000      //this many cycles are ignored
numMHIter        10        //This is for BayesB Metropolis Hastings
analysisType     Bayes     //Bayes, StepWise, Predict, generateData or LD
bayesType        BayesCCSub
probFixed        0.95
varGenotypic     .00063
varResidual      .0017     //This should be same as scaleRes (round 1)
subModelSize     100       // This is the number of markers in the Submodel
numIterSubModel  21000     // This is the number of iterations in the Submodel
```

Output from the BayesCCSub option is limited to the secondary or sub chain. An alternative approach to implement subset analyses is to run any bayesType other than BayesCCSub, then create a file containing just the list of interesting markers on the basis of information in the .mrkRes file. That filename can then be used following the keyword includeFileName to limit a subsequent analysis of any bayesType to just that subset of markers.

Output options for Bayes Analyses

In addition to brief screen output, and the information contained in the .out file, GenSel Bayesian analyses can create other output files, some of which are optional. All output files have the same filename as the .inp file such as .mrkResn or .cgrResn suffixes, where n is the number trailing the name of the .out file. The .cgrRes file outputs the names, levels and posterior effects for the fixed effects in the model, as specified in the model equation as defined in the phenotypic data file. Note the effects are not constrained to be full rank. The .mrkRes file outputs the results summarizing the fitting of each covariate from the genotypic file. An example is as follows

Marker	Effect	EffectVar	ModelFreq	GeneFreq	GenVar	EffectDelta1	SDDelta1	t-like	shrink
2	4.508e-05	9.064194e-07	0.1125	0.869	4.624949e-10	4.00737e-04	2.54964e-03	0.157	0.057
4	1.500e-04	1.198918e-06	0.1425	0.848	5.813457e-09	1.05275e-03	2.98090e-03	0.353	0.072
5	1.683e-05	8.959257e-07	0.1075	0.624	1.329147e-10	1.56521e-04	3.07546e-03	0.051	0.114
6	2.769e-05	1.123610e-06	0.1375	0.188	2.342253e-10	2.01418e-04	2.67184e-03	0.075	0.079
8	1.250e-04	1.111810e-06	0.1325	0.286	6.388826e-09	9.43684e-04	2.76849e-03	0.341	0.103
9	2.298e-05	8.770908e-07	0.1050	0.437	2.598589e-10	2.18888e-04	3.33920e-03	0.066	0.130
10	9.979e-05	9.568172e-07	0.1175	0.364	4.608782e-09	8.49267e-04	2.74301e-03	0.310	0.110
11	-1.565e-04	1.032000e-06	0.1250	0.652	1.110908e-08	-1.25202e-03	2.50209e-03	0.500	0.112
12	-1.269e-04	1.226026e-06	0.1475	0.274	6.413267e-09	-8.60407e-04	2.75417e-03	0.312	0.101
13	-3.066e-05	7.917254e-07	0.0950	0.089	1.527405e-10	-3.22767e-04	2.89318e-03	0.112	0.046

where the Marker defines the integer covariate titles that were used in the genotypic data file, and the Effect column defines the posterior mean of the covariate effect averaged across the post-burnin chain. In the case of analyses with pi greater than 0 these effects are averaged across the chains that include and exclude the particular effect in the model. The EffectVar defines the mean of the variance used for that locus, averaged across the post-burnin chains including the realizations that excluded that covariate from the model. The ModelFreq reflects the proportion of post-burnin realizations that included that particular covariate in the model. Generally, when pi >0 the model frequency will be highly correlated with the estimate of the absolute value of the effect, or the effect variance. The GeneFreq reflects the frequency of the fitted allele, rather than the minor allele frequency. GenVar, reflects the contribution of that locus to the genetic variance, calculated as 2p(1-p) times the square of the mean effects for that locus. Effectdelta1 reports the posterior mean effect for only those chains that included the effect in the model, ie Effect/ModelFreq. SDDelta1 is the standard deviation of the posterior distribution of effects for only those chains that included the effect in the model. The last column, t-like, is the ratio of the absolute value of EffectDelta1/SDDelta1. This latter statistic is highly correlated with ModelFreq but may have some additional utility in distinguishing informative markers with consistent versus inconsistent estimates of effects. The last column, shrink, reports the extent of shrinkage in the solution due to treating the effect as random in the mixed models relative to fitting in using least squares. This number is the ratio of the diagonal element of the coefficient matrix ignoring the variance ratio divided by the actual diagonal element of the coefficient matrix. The shrinkage varies by method and by the choice of pi. Shrinkage is reduced when there are more observations than when the dataset size is small. In models with pi=0, modelFreq will be constant for all markers. In BayesC analyses, EffectVar will be identical for all markers if pi=0, otherwise EffectVar will vary in those analyses due to the contribution

of ModelFreq on posterior variance averaged across the chain. In BayesCCSub analyses, only the subchain results will be reported.

Shrinkage in reported estimates of Effects (column 1 in .mrkRes) can originate from two sources, depending upon the BayesType. All effects will be shrunk to varying extents by the treatment of the effect as random. First, the amount of shrink varies with the variance ratio added to the diagonal (i.e. residual variance divided by effect variance), in relation to the amount of data (i.e. sums of squares of the covariate values). This shrinkage is reported in the trailing column of .mrkRes, and will be present in all BayesType analyses. Second, shrinkage may occur from Bayesian Variable Analysis when pi>0, due to variation in model frequency from one locus to the other. This will not occur in BayesA or BayesC with probFixed=0. The reported Effect is the posterior average, computed over chains with the effect in the model (i.e. EffectDelta1) in product with ModelFreq.

Detailed information as to the posterior distributions of the genetic variance, residual variance, phenotypic variance and proportion of phenotypic variation accounted for by markers can be output for every realization of the post-burnin chain using the keyword mcmcSamples yes in the .inp file. This option will create a text file ending in .mcmcSamplesn, containing four columns without a header. These columns are the genetic variance, residual variance, variance ratio and pi, respectively. The latter column will be fixed except in BayesCPi.

The information contained in the mcmcSamples file can be graphically displayed using the keyword plotPosteriors yes. This option will automatically set the mcmcSamples option to yes. This option requires that gnuplot is installed on the computer in a directory that is reflected in the environment variable. This might typically be /usr/local/bin or /opt/local/bin on Linux and Mac computers respectively. This option will produce post-script files terminating in resVarpltn.ps, genVarpltn.ps, h2pltn.ps for any of the Bayes methods. In the case of BayesCPi, the file ending in pipltn.ps will contain a histogram (see example below) of the posterior distribution of pi. On Linux computers a utility like ps2pdf will need to be installed to convert the postscript files to .pdf format. A utility such as xpdf can also be installed to allow the .pdf files to be visualized on the screen of a Linux machine.

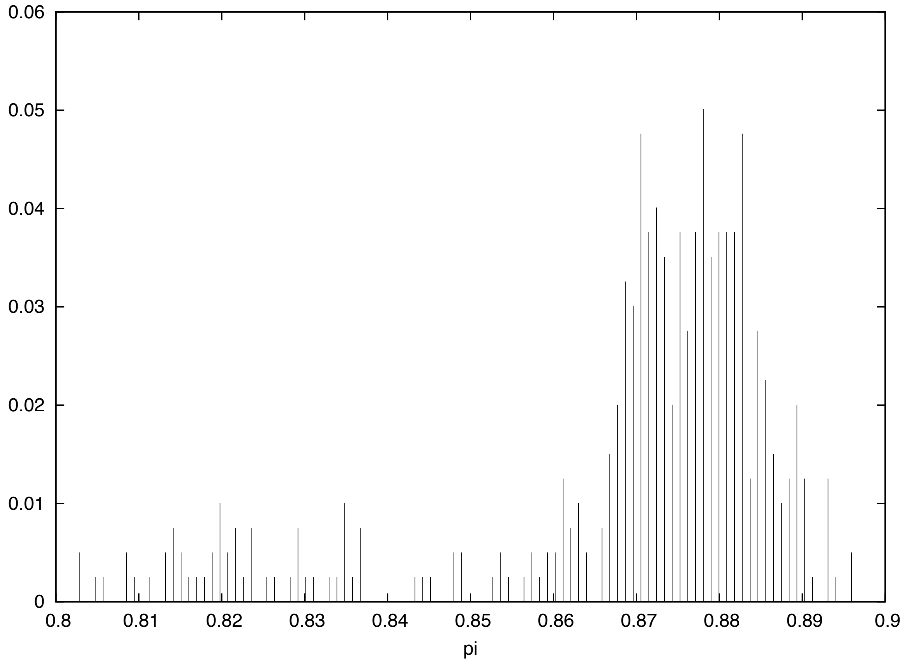

The keyword outputFreq can be added to the .inp file in order to dictate the frequency that summary information is written to the .out file. This can be useful for monitoring progress of an analysis.

The keyword modelSequence can be used in Bayes analyses with the option yes, to trigger the production of an output file with the suffix .modSeqn where n corresponds to the .out file digit designator. This large file will contain a list of the covariates that were included in the model at every step in the post burnin chain. It is of no value when pi=0 as all covariates will be in every model. The output file can be post processed to determine conditional frequencies of various markers in the model.

Pathogenic Datasets

Some analyses with BayesCPi or with pi very close to 1 and with data simulated for linkage studies (i.e. no founder LD) or with sparse marker densities may exhibit an almost complete lack of informative markers. In these cases, some chains may not include any loci, thereby contributing only to estimation of the residual variance. Accordingly, the posterior for the genetic variance and for the heritability will include considerable support for 0. In that situation, chains with no loci are discarded and the frequency of this occurrence is accumulated and reported as a warning in the .outfile. The number of chains fitted will be progressively increased so that the chainLength reflects the number of chains with at least one marker in the model.

Predicting Genomic Merit

Following the Bayes (or least squares) analyses, a frequently required option is to predict the genomic merit of individuals based on their genotypes and the posterior means (or least squares estimates) of the predicted effects that were output in the .mrkRes file. That analysis is undertaken using the Predict analysisType. In that event, the .inp file must include the name of the .mrkRes file to be used in the prediction, following the keyword markerSolFileName. It is important that the markers being used in the prediction align exactly with the markers in the genotype file. Accordingly, an includeFileName keyword will be required if a subset of the genotypes is being used in the prediction. The resultant solutions for animals included in the phenotype file will be output to a file with the suffix .ghatn. An example of that file follows.

```
Predict example

markerFileName        genotypes.test
phenotypeFileName     test.2marb
markerSolFileName     marbn2.mrkRes1
includeFileName       marbn2.100markers
analysisType          Predict
```

This would produce a .ghat file that might contain the following.

Animal ID	gHat	MARB	MARB_ACC#	grp#	sire$
1003	-7.38503411e-02	-0.03000000	0.13	1	7478280
1007	9.68837291e-02	-0.28999999	0.52	1	8989216
1008	1.00155063e-02	0.34999999	0.38	1	10383408
1009	-4.10633944e-02	-0.18000001	0.26	1	2467117

Note that the .ghat file contains the animal identifier, followed by the genomic prediction, and then the verbatim content of the phenotypic input file. This is useful as it provides the name of the trait corresponding to the genomic predictions, and the training data itself to facilitate computing correlations between predicted merit and breeding values of phenotypes for example for the purpose of cross validation.

Least Squares

A stepwise least squares procedure can be used to identify marker subsets. This procedure fits all the fixed effects dictated by the model equation defined in the phenotypic data file, and then undertakes forward and reverse stepwise regression. At each step, the covariate with the most significance will be added to the model, provided it excess the keyword alphaValue defined in the input file. After each marker is added to the model, a reverse stepwise procedure is implemented to ensure that all the markers currently in the model still exceed the alpha value. The stepwise procedure will terminate if either of the options defined by the keywords inputMaxRsquared or inputMaxMarkers are exceeded.

Example StepWise input file.

```
analysisType        StepWise
inputMaxRsquared    0.90
inputMaxMarkers     100
alphaValue          0.1
```

Simulating Data for Analysis

The keyword analysisType with the option generateData is used to simulate genotypes and phenotypes for analysis. This option requires the probFixed parameter to determine the proportion of loci that will be chosen at random and treated as causative. The keyword varGenotypic is used to determine the variance to be attributed to the gene effects that are drawn from a normal distribution for each of the randomly chosen causative loci. The keyword varResidual is used to determine the variance of residual effects to be added to the genotypes to simulate the phenotypes. Collectively these two keywords determine the heritability of the simulated trait.

The simulation option produces separate files for simulated genotypes and simulated phenotypes. The suffixes for these files are .GEffn and .simPhenotn, respectively, with n determined by the sequential number appropriate for the unique naming of the .out file.

generateData example (excluding marker and phenotype file definitions)

```
probFixed       0.95
analysisType    generateData //Bayes, StepWise, Predict, generateData or LD
varGenotypic    4.
varResidual     4.      //This should be same as scaleRes (round 1)
```

Linkage Disequilibrium

An important determinant of the accuracy ceiling for genomic prediction is dictated by the extent of linkage disequilibrium (LD). Linkage disequilibrium breaks down when two loci vary in allele frequency, but may also erode through recombination when allele frequencies remain similar in magnitude. It is often of interest to characterize the extent of LD in some or all markers used in genomic prediction. GenSel offers an option for computing LD using the keyword analysisType LD. The measure of LD is simply the square of the correlation between each pair of loci. The resulting output file of LD measures will be very large (several Gb) if applied to 50k loci. Analyses can be readily undertaken using a subset of markers through the include or exclude keywords. The output file ends in the suffix .LDn and contains the half-stored matrix of pair-wise squared correlations, sorted by column within row. The minor allele frequency of the row and column loci is also output, facilitating filtering of results in the output file, such as ignoring LD when either locus has a low minor allele frequency.

Keyword Summary

includeRange	100:500.9045:9432	//mutually exclusive options
includeFileName	existing filename	//mutually exclusive options
excludeFileName	existing filename	//mutually exclusive options
markerFileName	existing filename (ending in .bin for binary option)	
phenotypeFileName	existing filename	

alphaValue	0.1	//analysisType StepWise
analysisType	Bayes	//Bayes, StepWise, Predict, generateData or LD
bayesType	BayesA	//BayesA, BayesB, BayesC, BayesCPi, BayesCCSub.
Burnin	integer<chainLength	//this many cycles are ignored in Bayes
chainLength	integer	//this is total number of iterations in Bayes
degreesFreedomEffectVar	integer	//df for marker variance, typically 4 or 3
inputMaxRsquared	0.90	//analysisType StepWise
inputMaxMarkers	100	//analysisType StepWise
markerSolFileName	existing filename	//Predict
mcmcSamples	yes	//default typically no
modelSequence	yes	// default is no
numIterSubModel	integer	//BayesCCSub
numMHIter	integer	//This is for BayesB Metropolis-Hastings
nuRes	integer	// This is df for residual in Bayes
outputFreq	integer	//for Bayes chains
postPosteriors	yes	//requires gnuplot installed default is no
probFixed	0.95	//BayesB, BayesC, ByesCPi, BayesCCSub
seed	4567.5	//seed for random numbers default is 1234
subModelSize	integer	//BayesCCSub

varGenotypic	real	//Bayes
varResidual	real	//Bayes - this should be same as scaleRes (round 1)
